# Supplementary material for: Event-Related Potential to Conscious and Nonconscious Emotional Face Perception in Females with Autistic-Like Traits
Source: J Clin Med. 2020 Jul 21;9(7):2306. doi: 10.3390/jcm9072306 (PMC7408869; doi:10.3390/jcm9072306)
Supplement: Supplementary file 1 [file jcm-09-02306-s001.pdf]

Event-related potential to conscious and nonconscious emotional face perception in females with autistic-like traits

Vilfredo De Pascalis, Giuliana Cirillo, Arianna Vecchio and Joseph Ciorciari

Supplementary Statistical Results

Behavioral and Personality Results

Pearson correlation coefficients among personality traits of interest together with descriptive statistics are reported in Table 1. The correlation between RAPM and AQ was also not significant (Table 1).

The repeated measure ANCOVA on accuracy scores yielded a significant main effect for AQ ($F(1,48) = 25.82$, $p < 0.001$, $\eta^2_P = 0.349$), showing a lower accuracy in Hi-AQ scores compared to Lo-AQ ones (i.e., M = 71.0%, SD = 6.5 vs M = 76.5%, SD = 0.04). The main effect of Condition ($F(1,48) = 277.62$, $p < .001$, $\eta^2_P = 0.853$) and the AQ x Condition interaction ($F(1,48) = 7.93$, $p = 0.0137$, $\eta^2_P = 0.144$) were both significant. These effects indicated a lower accuracy rates in the subliminal compared to supraliminal condition (M = 55.3, SD = 7.0 vs M = 92.3%, SD = 7.5) and that the recognition accuracy of supraliminal faces in Hi-AQ was significantly lower than Lo-AQ participants (M = 88.2%, SD = 8.5% vs M = 96.3%, SD = 3.1, $p < 0.001$), while between group difference of subliminal faces did not reached the significance level (M = 53.8%, SD = 6.5% vs M = 56.8%, SD = 7.3, $p = 0.06$). Further, Facial-Gender factor was also significant ($F(1,48) = 23.83$, $p < 0.001$, $\eta^2_P = 0.331$) showing a higher accuracy rates for female than male faces that (M = 76.3%, SD = 4.5% vs M = 71.2%, SD = 10.1%). Additionally, the Facial-Gender x AQ interaction was highly significant ($F(1,48) = 61.18$, $p < 0.001$, $\eta^2_P = 0.560$). This effect disclosed that in high AQ participants accuracy to recognize facial expression with female faces was significantly higher than that with male faces (M = 76.5%, SD = 5.3, vs M = 65.6, SD = 10.5%, $p < 0.001$), while in Lo-AQ there were no differences in facial expression recognition with female versus male faces (M = 76.2, SD = 3.5, vs M = 76.9, SD = 5.5%, $p > 0.05$). This effect also indicated that Lo-AQ individuals had a significantly higher accuracy for male faces than Hi-AQ ones ($p < 0.001$), while for female faces there were no groups difference in accuracy ($p > 0.05$). Finally the forth order interactions of Facial-Gender x Condition x Emotion x AQ was significant ($F(2,96) = 2.99$, $p < 0.05$, $\eta^2_P = 0.057$). This interaction indicated that Hi-AQ participants had higher accuracy in detecting emotions of female faces than male faces for both subliminal and supraliminal stimuli. In contrast, in Lo-AQ participants no gender-face differences were observed (Figure 2a).

The repeated measures ANCOVA applied for the d′ scores revealed the main effect of Condition ($F(1,48) = 42.83$, $p < 0.001$, $\eta^2_P = 0.472$), indicating larger d′ values for supraliminal than subliminal condition (M = 4.53, SD = 1.62 vs M = 0.84, SD = 0.51). In addition, we found significant the effects of Emotion x Condition ($F(2,96) = 10.23$, $p < 0.001$, $\eta^2_P = 0.175$) and Condition x Emotion x Facial-Gender ($F(2,96) = 3.41$, $p < 0.05$, $\eta^2_P = 0.067$). The paired t-test for subliminal faces indicated significant differences between all emotions (happy: M = 0.506, SD = 0.496, neutral: M = 1.251, SD = 1.751, sad: M = 0.682, SD = 0.463), particularly showing smaller d′ values for happy compared to neutral ($p < 0.05$) and sad faces ($p < 0.05$) and larger d′ values for neutral compared to sad faces ($p < 0.05$) faces. Paired t-test for supraliminal showed higher d′ for happy than neutral faces (M = 4.772, SD = 2.017 vs M = 3.960, SD = 1.864, $p < 0.01$) and for sad than neutral faces (M = 4.852, SD = 2.153 vs M = 3.960, SD = 1.864, $p < 0.01$), while there were no differences between happy and sad faces (M = 4.772, SD = 2.017 vs M = 4.852, SD = 2.153, $p > 0.05$). The significant third-order interaction disclosed that for supraliminal sad stimuli d′ value was higher for male faces than female-faces ($p < 0.001$, Fig. 2b).

A similar ANCOVA on the response bias *c* values disclosed a significant effect of Condition ($F(1,48) = 50.63$, $p < 0.001$, $\eta^2_P = 0.513$), indicating larger c for subliminal than supraliminal condition

(M = 0.148, SD = 0.082 vs M = -0.012, SD = 0.014, respectively). The main effects of Emotion (F(2,96) = 13.53, $p < 0.001$, $\eta^2_p = 0.219$) and the interactions of Emotion x Condition (F(2,96) = 13.03, $p < 0.001$, $\eta^2_p = 0.214$) were also significant. The paired t-test revealed a smaller c for happy compared to neutral and sad faces (all $p < 0.001$). In addition, the significant triple interaction of Gender-Face x Emotion x Condition (F(2,96) = 3.65, $p < 0.05$, $\eta^2_p = 0.070$) indicated that in the subliminal condition, there was a smaller c for happy compared to neutral and sad faces (all $p < 0.001$, Fig. 2c). There were no significant differences among emotions in the supraliminal condition (all $p > 0.05$, Fig. 2c). These results indicate that participants were more likely to report happy faces than other faces in subliminal condition. The ANCOVAs performed on c values did not disclose any significant effect involving AQ trait.

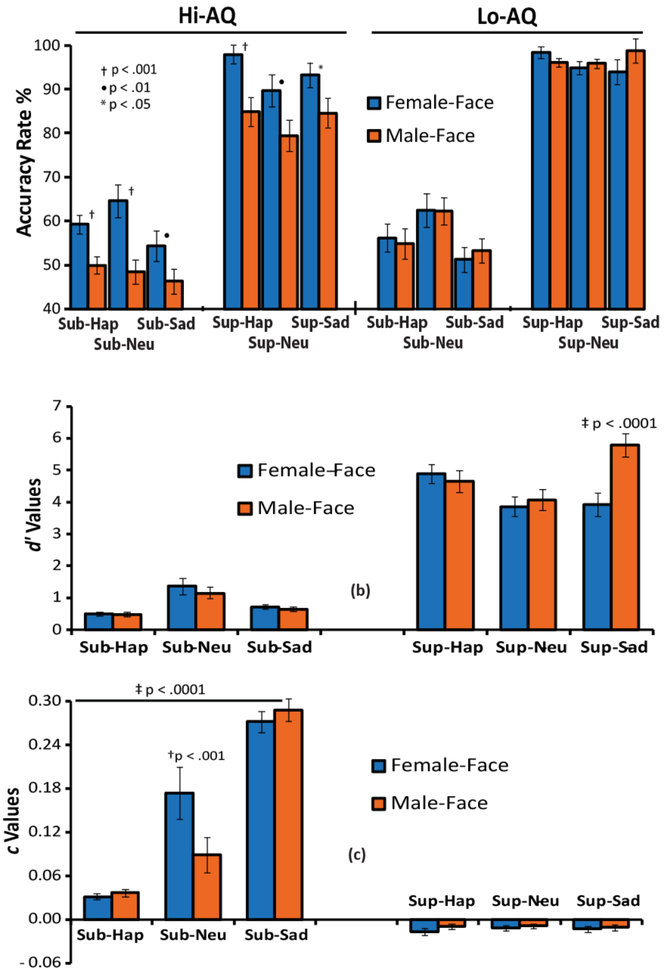

Figure 2s. (**a**) Mean performalece values of accuracy, (**b**) signal detection measure *d'* and (**c**)response bias *c* across subliminal and supraliminal stimuli for happy, neutral, and sad female- and male-faces.

ERP Results

N1 Amplitude and Latency

The ANCOVA, using AQ scores as a covariate, on the N1 amplitude data showed a significant main effect of Location (F(3,144) = 13.64, $p < 0.001$, $\eta^2_p = 0.221$), indicating that the N1 but was larger at frontal (Fz) than parietal (Pz) and occipital (Oz) regions (both $p < .0001$) and also larger at central (Cz) than Pz and Oz (both $p < .001$) regions. Further, the significant Emotion x Location interaction (F(2,288) = 5.11, $p = 0.0022$, $\eta^2_p = 0.096$) showed larger N1 amplitudes for happy than neutral and sad faces at Fz, Cz and Pz sites (Fz: M = 7.2, SD = 3.0; M = 3.6, SD = 1.5; M = 3.3, SD = 1.5; Cz: M = 6.6, SD = 3.2; M = 3.4, SD = 1.5; M = 3.4, SD = 1.5; Pz: M = 2.5, SD = 2.4; M =1.3, SD = 1.3; M = 1.3, SD = 1.1, respectively for happy, neutral, and sad faces; all ts, $p < 0.001$). In addition, the significant Emotion x Location x AQ interaction (F(6, 288) = 4.48, $p = 0.0046$, $\eta^2_p = 0.083$). Simple effect analysis at each recording site indicated that in the Lo-AQ group there was a larger negative peak for sad compared

to happy and neutral expressions at Pz, and P4 leads (Pz: sad vs happy t = 2.30, $p < 0.05$; sad vs neutral t = -2.21, $p < 0.05$; P4: sad vs happy t = 3.55, $p < 0.01$; sad vs neutral t = -2.17, $p < 0.05$; paired t-tests respectively for sad vs happy and sad vs neutral faces). In contrast paired samples t-tests performed separately in the Hi-AQ groups did not disclose any significant difference between emotion (all $p >$ 0.05; see right quadrant of Fig. 3).

The ANCOVA on N1 latency showed a main effect of Location (F(3,144) = 49.80, $p = 0.0019$, η^2_P = 0.509) and of Condition (F (1,48) = 6.65, $p = 0.015$, η^2_P = 0.122). The first effect showed a progressive significant reduction in N1 latency starting from Fz to Cz, Pz, and Oz sites (all $p < 0.001$). The second main effect indicated that subliminal stimuli elicited shorter N1 latencies than supraliminal stimuli (Table 2). Moreover, the interaction effect of AQ x Emotion was also significant (F(2,96) = 4.86, p = 0.0123, η^2_P = 0.092), showing that in Hi-AQ participants happy faces had a longer N1 latency than neutral and sad faces (M = 106, SD = 4.5 vs M = 104, SD = 4.6, $p < 0.05$ and vs M = 104, SD = 4.9, $p <$ 0.05), while there were no differences among emotional faces in Lo-AQ (M = 102, SD = 6.2 vs M = 103, SD = 6.2, and M = 103, SD = 6.2, all $p > 0.05$). In addition, Hi-AQ had a longer N1 latency to happy faces compared to Lo-AQ participants (M = 106, SD = 4.5 vs M = 102, SD = 6.2, $p < 0.05$), while there were no latency differences between AQ groups for the neutral and sad faces. Further analysis found the main effect of AQ (F(1,48 = 4.95, $p = 0.031$, η^2_P = 0.093) in the occipital (Oz) region,indicating a significantly relatively longer N1 latency in the Hi-AQ compared to the Lo-AQ scorers. This finding was in support of our second main hypothesis and in line with Fujita, Yamasaki, Kamio, Hirose, and Tobimatsu (2011) findings in ASD patients. Finally, the interactive effect of Facial-Gender x Emotion x Location, was significant (F(6,288) = 4.91, $p < 0.001$, η^2_P = 0.093) as well as the Facial-Gender x Emotion x Location x AQ (F(6,288) = 3.10, $p = .0123$, η^2_P = 0.061). These effects disclosed that for female happy faces HiAQ had a longer N1 latency than Lo-AQ scorers at Fz and Oz scalp leads, while for female sad faces this between group differences was significant for the only Fz lead. For male happy and sad faces we also found a relative longer N1 latency in Hi-AQ scorers, although this difference was significant at only the occipital lead. These effects are displayed in Figure 4 (see text)

VPP Amplitude and Latency

The analysis of the VPP amplitude showed a Condition main effect (F(1,48)= 4.98, $p = 0.0331$, η^2_P = 0.094). This effect showed a larger VPP amplitude to supraliminal than subliminal faces (Fig. 4a). The Location effect was significant (F (1,48) = 7.62, $p < 0.001$, η^2_P = .137) showing a smaller P2 at Fz compared to Cz, Pz and Oz sites with Oz showing the largest VPP peak (all $p < 0.001$). In addition, the Condition x Emotion was also significant (F(2,144) = 12.37, $p < 0.001$, η^2_P = 0.204). The pairwise comparison showed within supraliminal stimuli a larger VPP to happy than neutral faces ($p < 0.01$) and to sad than neutral faces ($p = 0.001$; Fig. 4b). In contrast, for subliminal faces we had a significantly smaller VPP to happy compared to neutral and sad than neutral (both $p < 0.05$) , while there was a larger VPP amplitude to neutral faces than sad faces ($p < 0.001$; Fig. 4b).

The ANCOVA for VPP latency found a significant main effect of Condition (F (1,48) = 7.30, p = 0.0123, η^2_P = 0.132) disclosing that subliminal stimuli elicited shorter VPP latencies than supraliminal stimuli (Tab. 2). Additionally, the effect for Location was significant (F(3,144) = 55.71, $p < 0.001$, η^2_P = 0.537) that showed a progressive significant reduction in VPP latency from Fz and Cz to Pz, and Oz regions (all $p < 0.01$).

Table 1. N170 mean latency (M) and standard deviation (SD). collapsed across T5 and T6 scalp leads for Happy, Neutral and Sad facial expressions in the Supraliminal and Subliminal conditions

Facial Expressions	M	SD
Happy Supraliminal	176.5	13.6
Happy Subliminal	184.4	15.9
Neutral Supraliminal	177.7	17.8
Neutral Subliminal	178.3	14.2
Sad Supraliminal	180.3	15.4
Sad Subliminal	186.8	19.8

N170 Amplitude and Latency

The analysis on the N170 amplitude data showed a significant effect of Condition ($F(1,48) = 7.99$, $p = 0.0123$, $\eta^2_P = 0.142$), which was due to a larger N170 peak to supraliminal than subliminal faces (left and right quadrants of Fig. 5a) . Moreover, the Condition by Emotion interaction was also significant ($F(2,96) = 6.59$, $p = 0.0034$, $\eta^2_P = 0.120$), and further analysis indicated a significantly smaller N170 under subliminal condition to sad faces than happy and neutral faces ($p < 0.01$; Fig. 5b).

The analysis of N170 peak latencies revealed that Emotion x Contition ($F(2, 96) = 3.69$, $p = 0.029$, $\eta^2_P = 0.071$) was significant. This effect showed that the N170 latency of both happy and sad expressions (but not neutral) was sorter in the supraliminal condition than in the subliminal one (see Table S1; $p < 0.001$, $p = 0.765$, and $p = 0.0131$, respectively for happy, neutral and sad faces). Moreover, the interaction of AQ x Facial Gender was significant ($F(1,48) = 8.31$, $p = 0.0086$, $\eta^2_P = 0.147$). This effect showed that in Hi-AQ participants there were no differences on N170 latency between female and male faces (M = 181.1, SD = 13.7 vs M = 182.3, SD = 12.9, $p > 0.05$), while in Lo-AQ participant female faces had longer latency than male faces (M = 180.6, SD = 14.8 vs M = 176.1, SD = 10.5, $p < 0.05$).

N2 Amplitude and Latency

The analysis of the midline N2 amplitudes yielded the significant interactions of Facial-Gender x Emotion ($F(2, 96) = 8.00$, $p = .0025$, $\eta^2_P = 0.143$) and of Facial-Gender x Emotion x Location ($F(6, 288) = 6.08$, $p < 0.001$, $\eta^2_P = 0.114$). These effects indicated that for happy female-faces there was a larger frontocentral N2 than for male-faces, while for sad faces there was an opposite trend between female and male faces (all $p < 0.05$; Fig. 6).

The ANCOVA for N2 latency found a significant main effect of Location ($F(3,144) = 62.52$, $p < 0.001$, $\eta^2_P = 0.57$) and of Condition ($F(1, 48) = 35.76$, $p < 0.001$, $\eta^2_P = 0.427$). The Location effect showed a progressive significant reduction in N2 latency from Fz and Cz to Pz and Oz regions (all $p < 0.001$). The Condition effect indicated that subliminal stimuli elicited shorter N2 latencies than supraliminal stimuli (M = 225, SD = 10.8 vs M = 209, SD = 10.1, $p < 0.01$). No other main or interaction effects were significant.

P3 Amplitude and Latency

The P3 amplitude analysis yielded a significant Location effect ($F(3,144) = 9.45$, $p = 0.0019$, $\eta^2_P = 0.164$) showing larger P3 waves in the Pz and Oz regions than Fz and Cz regions (Fz: M = -0.74, SD = 1.54; Cz: M = 1.32, SD = 1.82; Pz: 3.01, SD = 1.76; Oz: M = 5.43, SD = 3.22; all $p < 0.001$). Further, the following interaction were all significant: Emotion x Location ($F(6,288) = 4.59$, $p= 0.0022$, $\eta^2_P = 0.087$), Emotion x Location x AQ ($F(6, 288) = 3.22$, $p = 0.0147$, $\eta^2_P = 0.062$) and Facial-Gender x Emotion x Location x AQ ($F(6, 288) = 3.58$, $p = 0.0032$, $\eta^2_P = 0.069$). The first interaction showed a larger occipital P3 to sad faces than neutral and happy faces (M = 5.03, SD = 3.3 vs M = 5.19, SD = 3.4, $p > 0.05$; M = 5.19, SD = 3.4 vs M = 6.05, SD = 3.27, $p < 0.05$; M = 5.03, SD = 3.3 vs M = 6.05, SD = 3.27, $p < 0.01$; repectively for happy vs neutral, neutal vs sad and happy vs sad faces). The second interaction disclosed that Hi-AQ had a larger P3 at occipital lead to sad faces than Lo-AQ (M = 3.4 SD = 1.4 vs M = 2.2, SD = 1.5, t = 2.92, $p < 0.01$; respectively). The last interlinked effects indicated that for male happy and sad faces Hi-AQ participants elicited a larger parietal occipital P3 than Lo-AQ ones, while difference between AQ groups was significant for sad female faces alone (all $p < 0.01$; see Fig. 7).

The P3 latency analysis showed a main effect of Condition ($F(1,48) = 20.48$, $p < 0.001$, $\eta^2_P = 0.300$), indicating significantly shorter P3 latencies in the subliminal than supraliminal condition (M = 288, SD = 12.4 vs M = 309, SD = 13.8). The Location main effect was significant ($F(3,144) = 56.15$, $p < 0.001$, $\eta^2_P = 0.539$). The Location effect indicated that P3 latencies in Fz and Cz regions were significantly longer than those in Pz and Oz, as well as that in Pz than Oz (Table 2), all $p < 0.001$). The Condition effect showed that there was a robust P3 latency reduction in subliminal compared to supraliminal condition (Table 2).

N4 Amplitude and Latency

There were no significant main effects for N4 amplitudes, with the exception of Location ($F(3,144) = 9.68$, $p < 0.001$, $\eta^2_P = 0.168$), showing larger N4 wave in Fz and Oz regions.

The analysis for the N4 latency found a significant Facial-Gender main effect ($F(1,48) = 6.08$, $p = 0.020$, $\eta^2_P = 0.112$), and a significant interaction Facial-gender x Location interaction ($F(3, 144) = 9.11$, $p = 0.0007$, , $\eta^2_P = 0.159$) and indicated a significantly shorter N4 wave to female faces than male-faces in Pz and Oz recordings (Pz : M = 384, SD =16.3 vs M = 388, SD = 11.5, $p < 0.05$; Oz: M = 350, SD = 28.5 vs M = 364, SD = 26.8, $p < 0.05$; respectively for female faces vs male-faces). The Emotion main effect was also significant ($F(2, 96) = 4.83$, $p = 0.0131$, $\eta^2_P = 0.091$) and disclosed a longer N4 latency to sad faces than happy and neutral faces (M = 384.3, SD = 9.8 vs M = 378.2, SD = 11.5 and M = 376.1, SD = 10.2; respectively, both ps < 0.05).
